# Detección de *Aedes* (*Stegomyia*) *albopictus* (Skuse) en ovitrampas en Mérida, México

**DOI:** 10.7705/biomedica.5525

**Published:** 2020-08-28

**Authors:** Gabriela González-Olvera, Magally Morales-Rodríguez, Wilbert Bibiano-Marín, Jorge Palacio-Vargas, Yamili Contreras-Perera, Abdiel Martín-Park, Azael Che-Mendoza, Marco Torres-Castro, Fabián Correa-Morales, Herón Huerta-Jiménez, Pedro Mis-Ávila, Gonzalo Vázquez-Prokopec, Pablo Manrique-Saide

**Affiliations:** 1 Unidad Colaborativa para Bioensayos Entomológicos, Campus de Ciencias Biológicas y Agropecuarias, Universidad Autónoma de Yucatán, Mérida, México; 2 Servicios de Salud de Yucatán, Mérida, México; 3 Laboratorio de Enfermedades Emergentes y Reemergentes, Centro de Investigaciones Regionales “Dr. Hideyo Noguchi”, Universidad Autónoma de Yucatán, Mérida, México; 4 Centro Nacional de Programas Preventivos y Control de Enfermedades, Secretaría de Salud, Ciudad de México, México; 5 Instituto de Diagnóstico y Referencia Epidemiológicos), Secretaría de Salud, Ciudad de México, México; 6 Secretaría de Salud de Quintana Roo, Chetumal, México; 7 Department of Environmental Sciences, Emory University, Atlanta, GA, USA

**Keywords:** *Aedes*, mosquitos vectores, control de vectores, enfermedades transmitidas por vectores, México, *Aedes*, mosquito vectors, vector control, vector borne diseases, México

## Abstract

**Introducción:**

El programa de enfermedades transmitidas por vectores en México tiene una red establecida de ovitrampas para la vigilancia entomológica de *Aedes* spp. Los servicios de salud del estado de Yucatán, en respuesta a reportes de *Aedes albopictus* en la periferia de Mérida, capital del estado, incrementaron la especificidad de dicha vigilancia.

**Objetivo:**

Describir la presencia y distribución de *Ae. albopictus* en Mérida y su abundancia relativa comparada con *Aedes aegypti*, en ovitrampas del programa de control de vectores.

**Materiales y métodos:**

Durante octubre de 2019, se seleccionaron al azar 91 ovitrampas en 31 barrios de Mérida. Los mosquitos adultos se obtuvieron del insectario de la Unidad Colaborativa para Bioensayos Entomológicos de la Universidad Autónoma de Yucatán a partir de huevos recolectados en campo. Se determinó la abundancia relativa de individuos adultos de cada especie identificada y por barrios evaluados.

**Resultados:**

En el 32% de los barrios muestreados, se detectó *Ae*. *albopictus* y, en todos ellos, *Ae*. *aegypti*. Se recolectaron 28 adultos de *Ae*. *albopictus* (10 hembras y 18 machos) en las ovitrampas. No se observó correlación entre la abundancia de adultos ni de hembras *Ae*. *aegypti* y *Ae*. *albopictus* por barrio (p>0,05).

**Conclusiones:**

Los resultados confirmaron que *Ae*. *albopictus* estaba coexistiendo con *Ae*. *aegypti* en Mérida en el momento del estudio. La baja abundancia relativa sugiere que *Ae*. *albopictus* se encontraba en la fase inicial de invasión.

## Abstract (en)

**Introduction:**

The vector-borne diseases program in México has an established network of ovitraps for entomological surveillance of *Aedes* spp. In response to reports of *Aedes albopictus* in the periphery of Mérida, the state capital of Yucatán, the Ministry of Health increased the specificity of this surveillance.

**Objective:**

To describe the presence and distribution of *Ae*. *albopictus* in Mérida and its relative abundance compared to *Aedes aegypti* in ovitraps of the vector control program.

**Materials and methods:**

During October, 2019, 91 ovitraps were randomly selected from 31 neighborhoods of Mérida. Mosquitoes were reared at the insectary of the Collaborative Unit for Entomological Bioassays of the Autonomous University of Yucatán from eggs collected in the field. Relative abundance was determined for adult individuals of each identified species and neighborhood.

**Results:**

32% of the neighborhoods were positive for *Ae*. *albopictus* and 100% for *Ae*. *aegypti*. A total of 28 adults of *Ae*. *albopictus* (10 females and 18 males) were obtained from ovitraps. No correlation was observed between the abundance of *Ae*. *aegypti* and *Ae*. *albopictus* for both adults and females (p>0.05) at the neighborhood level.

**Conclusions:**

The results confirm that *Ae*. *albopictus* coexisted with *Ae*. *aegypti* in Mérida at the time of the study. The low relative abundance suggests that *Ae*. *albopictus* was in the initial phase of invasion.

*Aedes* (*Stegomyia*) *albopictus* (Skuse), conocido comúnmente como “mosquito tigre asiático”, se considera un vector biológico secundario de algunos virus que ocasionan arbovirosis febriles de gran importancia en salud pública, como la fiebre por dengue y la fiebre por chikunguña (1), y también, es un vector competente para la transmisión del virus del Zika (2). Si bien es originario de Asia, en las últimas décadas ha invadido y se ha adaptado en extensiones grandes de las Américas, Europa y África (3,4).

En México, la presencia de *Ae*. *albopictus* se reportó por primera vez en 1998 en el noreste del país (5,6) y, desde entonces, se ha ampliado su distribución, particularmente en el sureste del país, donde existen las condiciones macroambientales y microambientales favorables para su establecimiento y supervivencia (7). En este contexto, en el 2018 se reportó la presencia de *Ae*. *albopictus* en la zona suburbana (periferia) de Mérida, capital del estado de Yucatán (8). Antes de este registro, en Yucatán únicamente se había reportado este mosquito en el municipio de Tizimín, 170 km al este de Mérida (9).

Aunque se desconoce la contribución real de *Ae*. *albopictus* en la transmisión de arbovirus en México, en los estudios realizados durante un brote de fiebre por dengue, se reportó la infección natural de esta especie con el virus del dengue, lo que sugiere que podría actuar como vector en las áreas rurales y urbanas del país (10). Su presencia en la periferia de Mérida, uno de los centros demográficos de México más importantes en términos epidemiológicos para la transmisión de los virus del dengue, chikunguña y Zika (11), puso en alerta a las autoridades locales de salud.

El programa nacional de enfermedades transmitidas por vectores de la Secretaría de Salud de México estableció la vigilancia entomológica con ovitrampas desde el 2009 (12). Actualmente, dicha vigilancia abarca 712 centros urbanos de diferente extensión y número de habitantes en los 32 estados del país. De esta forma, el sistema cuenta con la capacidad de detectar la presencia de los mosquitos *Ae*. *aegypti* y *Ae*. *albopictus* a lo largo del territorio nacional (12).

Mediante este sistema y en respuesta a la detección de *Ae*. *albopictus* en la periferia de Mérida (8), los Servicios de Salud de Yucatán incrementaron la especificidad de la vigilancia entomológica con ovitrampas para detectar *Ae*. *albopictus* en el interior de la ciudad, lo que hasta ese momento no se había reportado.

El objetivo del presente estudio fue describir la presencia y distribución actual de *Ae*. *albopictus* en Mérida, Yucatán, y su abundancia relativa comparada con *Ae*. *aegypti*, mediante ovitrampas del sistema de vigilancia entomológica de los servicios de salud locales.

## Materiales y métodos

La vigilancia entomológica en Mérida se basa en un sistema de 5.183 ovitrampas distribuidas en 225 barrios y fraccionamientos, las cuales se examinan semanalmente durante todo el año. La información resultante sirve para determinar la presencia de mosquitos *Aedes* spp. y su abundancia espacio-temporal para establecer el riesgo entomológico y dirigir o evaluar las intervenciones para su control integral (12-14). Los huevos recolectados de las ovitrampas también proveen material para el desarrollo de pruebas de eficacia biológica y de sensibilidad o resistencia a insecticidas en larvas y adultos, como parte del Sistema Nacional de Vigilancia de la Resistencia a cargo de las Unidades de Investigación Entomológica y Bioensayos estatales (15).

Como todos los de México, el programa de control de vectores de los servicios de salud de Yucatán, utiliza ovitrampas estandarizadas que consisten en un recipiente cilíndrico de plástico de color negro de un litro de capacidad recubierto en su tercio superior con una franja de tela pellón (F-1600), conocida como “papeleta”, que sirve como anclaje para la oviposición (12). El recipiente se coloca con agua (3/4 de su capacidad) en el peridomicilio (exterior) de las viviendas. En las ciudades, se recomienda instalar cuatro ovitrampas distribuidas en una manzana (grupo de bloques de pisos y casas rodeados por cuatro calles), espaciadas cada 4 a 6 manzanas para cubrir completamente la extensión urbana (12).

En este estudio, se seleccionó una muestra de 91 ovitrampas repartidas en 31 barrios de Mérida (tres ovitrampas por barrio), las cuales presentaban la mayor abundancia de huevos por ovitrampa en el período del estudio. La revisión de las ovitrampas se hizo del 21 al 31 de octubre de 2019. Los huevos de mosquito depositados en las papeletas fueron recolectados en campo por personal de los servicios locales de salud, siguiendo las directrices para embriogénesis, almacenamiento y envío de papeletas establecidos en la guía metodológica de vigilancia entomológica con ovitrampas del Centro Nacional de Programas Preventivos y Control de Enfermedades (12). El material se trasladó a la Unidad Colaborativa para Bioensayos Entomológicos de la Universidad Autónoma de Yucatán, que funciona como unidad de investigación entomológica y bioensayos de los servicios de salud de Yucatán.

La emergencia de los mosquitos adultos se llevó a cabo en condiciones de insectario: 26 °C ± 2 °C, 75% ± 5% de humedad relativa, y 12 horas de luz:12 horas de oscuridad. Cada papeleta se colocó en una charola plástica con dos litros de agua y las larvas se alimentaron con una mezcla de harina de carne y levadura (80:20). Los adultos emergidos se congelaron para su posterior identificación. Para fines de control de calidad, una muestra de especímenes se envió al Centro Nacional de Referencia del Instituto de Diagnóstico y Referencia Epidemiológicos de la Secretaría de Salud de México.

La abundancia relativa se determinó contabilizando los mosquitos adultos pertenecientes a cada especie, emergidos en las condiciones de insectario a partir de las papeletas recolectadas. La abundancia de *Ae*. *aegypti* y la de *Ae*. *albopictus* se compararon por barrio, las cuales no tienen una distribución normal, y se exploraron con una prueba de correlación *S* de Spearman en el programa R (versión 3.5.0). Se calculó la concordancia espacial en la distribución de *Ae*. *aegypti* y *Ae*. *albopictus* mediante el índice multivariado C de Geary (16), el cual permite determinar si los valores altos de una variable (abundancia de *Ae*. *aegypti*) se asocian con los de otra variable (abundancia de *Ae*. *albopictus*) considerando cada barrio y sus vecinos inmediatos mediante una función de vecindad del tipo reina (*queen*) (16). El índice C de Geary se calculó con el programa GeoDA (http://geodacenter.github.io/). Para todos los análisis, se estableció un valor alfa de significación de 5%.

## Resultados

La emergencia de adultos a partir de las pupas obtenidas en el insectario fue del 100%. De los 31 barrios estudiados, 10 (32,2%) fueron positivos para adultos de *Ae*. *albopictus*. Se obtuvieron hembras de *Ae*. *albopictus* en seis (19,4%) de los barrios y, machos, en 8 (25,8%). Todos los barrios fueron positivos para hembras y machos adultos de *Ae*. *aegypti* (cuadro 1).

**Cuadro 1:** Número de adultos de *Aedes aegypti* y *Aedes albopictus* emergidos de ovitrampas expuestas entre el 21 y el 31 de octubre de 2019 en barrios y fraccionamientos de Mérida, Yucatán, México

**Barrio**	**Total de huevos**	**Total** ***Ae*. *aegypti* emergidos**	**Hembras** ***Ae*. *aegypti* emergidos**	**Machos** ***Ae*. *aegypti* emergidos**	**Total** ***Ae*. *albopictus* emergidos**	**Hembras** ***Ae*. *albopictus* emergidos**	**Machos** ***Ae*. *albopictus* emergidos**
Azcorra	869	116	54	62	1	0	1
Bojórquez	1.279	54	19	35	0	0	0
Bosques del Poniente	790	30	15	15	0	0	0
Cámara de la Construcción	370	24	17	7	0	0	0
Centro	1.045	79	40	39	0	0	0
Chuburná de Hidalgo	341	26	10	16	0	0	0
Cinco Colonias	525	92	36	56	2	0	2
Felipe Carrillo Puerto	1.269	66	33	33	0	0	0
Fraccionamiento Paseo de las Fuentes	608	46	22	24	3	1	2
Fraccionamiento Mulsay	211	39	20	19	0	0	0
Fraccionamiento Polígono 108	1.326	60	19	41	4	1	3
Francisco I. Madero	309	48	22	26	0	0	0
García Ginerés	1.408	23	16	7	0	0	0
López Mateos	342	58	19	39	0	0	0
María Luisa	437	24	14	10	0	0	0
Miguel Alemán	378	52	18	34	0	0	0
Miraflores	987	35	24	11	3	3	0
Morelos Oriente	359	29	10	19	0	0	0
Nora Quintana	481	18	9	9	0	0	0
Nueva Kukulkán	314	53	23	30	1	1	0
Predio San José Tecoh	202	54	32	22	5	2	3
Reparto Granjas	331	25	12	13	0	0	0
Salvador Alvarado Sur	1.820	42	16	26	7	2	5
San Antonio Xluch	762	65	30	35	1	0	1
San José	456	56	24	32	1	0	1
San Pedro Uxmal	396	55	29	26	0	0	0
Serapio Rendón	345	127	60	67	0	0	0
Terranova	949	44	19	25	0	0	0
Xcumpich	738	36	20	16	0	0	0
Yucalpetén	1.906	52	22	30	0	0	0
Zapata Sur 3	691	41	24	17	0	0	0

Se obtuvieron 28 adultos de *Ae*. *albopictus* (10 hembras y 18 machos) en condiciones de insectario, lo que sugiere una abundancia mucho menor en comparación con los especímenes de *Ae*. *aegypti* recolectados (728 hembras y 841 machos).

**Figura 1:**
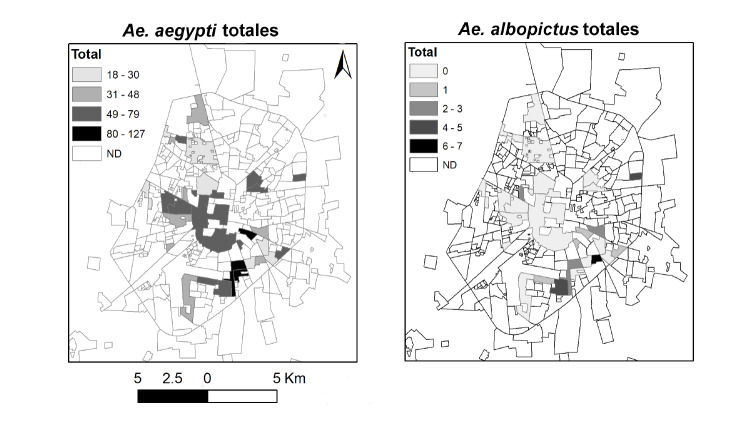
Distribución de *Aedes aegypti* y *Aedes albopictus* (adultos) obtenidos a partir de recolecciones con ovitrampas expuestas entre el 21 y el 31 de octubre de 2019 en barrios y fraccionamientos de Mérida, Yucatán, México

**Figura 2:**
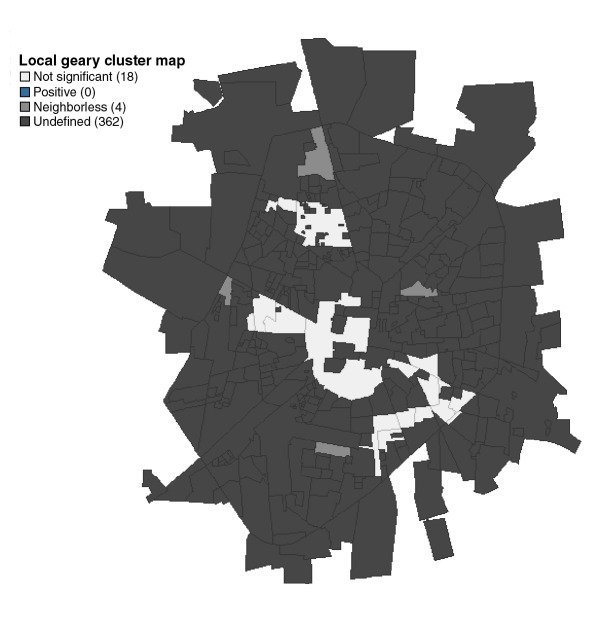
Resultados del estadístico espacial C de Geary para cuantificar la asociacion entre la abundancia de *Aedes aegypti* y *Aedes albopictus* por barrios, y fraccionamientos, considerando la distribucion de los barrios vecinos

La figura 1 muestra la distribución de los barrios positivos para cada especie de mosquito (*Ae*. *aegypti* y *Ae*. *albopictus*) y su abundancia total. No se observó una correlación entre la abundancia relativa de *Ae*. *aegypti* por barrio y la de *Ae*. *albopictus*, ni en el total de adultos (*S*=3329,9, p=0,07105) o el de hembras (*S*=4472,1; p=0,5985). El índice C de Geary no mostró una asociación significativa entre los valores de abundancia de *Ae*. *aegypti* y de *Ae*. *albopictus* por barrio (C>1,96; p>0,05) al considerar también las abundancias en barrios vecinos (figura 2).

## Discusión

Los primeros reportes de la presencia de *Ae*. *albopictus* en México provenían de la frontera norte entre 1988-1994 (5,6,17). Desde entonces, su presencia se ha descrito hasta el extremo sur del país en la frontera con Guatemala en el 2002 (18) y, más recientemente, en la península de Yucatán, en los estados de Quintana Roo en el 2012 (19) y Campeche en el 2019 (20). En el 2017, se detectó por primera vez en el nororiente del estado de Yucatán (9) y, dos años después, en los barrios y fraccionamientos alrededor de Mérida (8).

Los resultados del presente estudio señalan que *Ae*. *albopictus* está presente y coexiste con *Ae*. *aegypti* en el interior (zona urbana) de Mérida. La presencia focalizada de *Ae*. *albopictus* en Mérida y los reportes recientes de su distribución en el interior de Yucatán y en la periferia de Mérida (8,9), sugieren que este mosquito se halla en la fase inicial de invasión de la ciudad.

Se requieren estudios posteriores para establecer interacciones biológico-ecológicas, tales como la segregación o desplazamiento entre ambas especies de *Aedes.* La segregación en diferentes hábitats es uno de los mecanismos más comunes que favorecen la coexistencia de ambas especies y evita la competencia directa (21), tal y como ocurre en Florida, Estados Unidos, donde *Ae*. *aegypti* habita zonas más urbanizadas y, *Ae*. *albopictus*, zonas con mayor cobertura vegetal (4). En México, se ha encontrado *Ae*. *albopictus* coexistiendo en poca abundancia con *Ae*. *aegypti*, frecuentemente en zonas suburbanas de abundante vegetación, clima húmedo y cálido, y una gran disponibilidad de criaderos naturales y artificiales (9).

La distribución de *Ae*. *albopictus* reportada en México actualmente incluye 14 estados: Campeche, Ciudad de México, Coahuila, Chiapas, Hidalgo, Morelos, Nuevo León, San Luis Potosí, Sinaloa, Tabasco, Tamaulipas, Veracruz, Yucatán y Quintana Roo (8,9,17-24). Estos reportes, con excepción de los de Ciudad de México (23) y Morelos (24), provienen de estudios en que se han recolectado larvas e individuos adultos, por lo que en ninguno se ha descrito la detección de *Ae*. *albopictus* en la red de ovitrampas de programas estatales de vigilancia entomológica. Más aún, el presente trabajo representa el primer esfuerzo por cuantificar la proporción de adultos emergidos de *Ae*. *aegypti* y *Ae*. *albopictus* a partir de las ovitrampas de la red nacional de vigilancia entomológica.

En la actualidad, la Guía Metodológica para la Vigilancia Entomológica con Ovitrampas en México (12) establece que puede extraerse información específica para ambas especies de *Aedes*; no obstante, en la práctica, toda la información se relaciona con *Ae*. *aegypti*. Asimismo, la plataforma de vigilancia y control integrado del vector del Programa Nacional (http://kin.insp.mx/aplicaciones/EntomologiayControlIntegral/login.aspx), la cual recibe la información procedente de la red compuesta por 250.000 ovitrampas distribuidas en todo el territorio nacional, únicamente contiene información sobre *Ae*. *aegypti*. La inclusión de información específica sobre *Ae*. *albopictus* permitiría elaborar acciones específicas para la especie, con el fin de evitar o disminuir la propagación y adaptación de esta especie a nuevas áreas urbanas.

Dado que ambas especies de mosquitos (*Ae*. *aegypti* y *Ae*. *albopictus*) se detectaron en las ovitrampas y coexisten en determinadas áreas, su contribución a la transmisión de arbovirus puede diferir (25,26), por lo que es necesario validar los índices de ovitrampas (actualmente genéricos para *Aedes*) y las medidas de riesgo entomológico y epidemiológico.

Las ovitrampas son indudablemente una herramienta importante para los programas de control de mosquitos vectores de virus como el dengue, el chikunguña y el Zika. Sin embargo, una de sus limitaciones es la baja especificidad en presencia de varias especies de *Aedes,* por lo que es necesario incubar los huevos y criar las larvas en ambientes controlados (insectarios) para la identificación de las especies de mosquitos adultos, lo que requiere inversiones en tiempo y recursos. El bajo porcentaje de larvas emergidas con respecto al número total de huevos puestos a eclosionar en este estudio, es otro aspecto que debe mejorarse en la vigilancia de mosquitos *Aedes* con ovitrampas. En este sentido, se ha sugerido el uso de soluciones a base de levadura para aumentar la eficiencia de eclosión de huevos del género *Aedes* (27).

Por otra parte, en lo que respecta al control de las poblaciones de mosquitos vectores, la aplicación de adulticidas puede ser genérica o diferenciada según la biología de cada especie. En este contexto, la aplicación de adulticidas en exteriores comparada con la de interiores puede hacerse de forma selectiva, ya que se ha reportado que *Ae*. *albopictus*, por ejemplo, tiene preferencia por sitios de reposo ubicados en el peridomicilio, en tanto que *Ae*. *aegypti* prefiere sitios de reposo en el interior de las viviendas (28).

Los hallazgos de este estudio pueden servir para que los expertos, los programas locales y la Secretaría de Salud de México, discutan y planifiquen las acciones futuras de vigilancia y control genéricas de *Aedes* o específicas para *Ae*. *aegypti* y *Ae*. *albopictus*.
